# Plasma Sequencing for Patients with GIST—Limitations and Opportunities in an Academic Setting

**DOI:** 10.3390/cancers14225496

**Published:** 2022-11-09

**Authors:** Johanna Falkenhorst, Susanne Grunewald, Dawid Krzeciesa, Thomas Herold, Julia Ketzer, Miriam Christoff, Rainer Hamacher, Karina Kostbade, Jürgen Treckmann, Johannes Köster, Farhad Farzaliyev, Benjamin Samulon Fletcher, Nils Dieckmann, Moritz Kaths, Thomas Mühlenberg, Hans-Ulrich Schildhaus, Sebastian Bauer

**Affiliations:** 1 Department of Medical Oncology, Sarcoma Center, West German Cancer Center, University Hospital Essen, University Duisburg-Essen, 45147 Essen, Germany; 2 German Cancer Consortium (DKTK), Partner Site University Hospital Essen, 45147 Essen, Germany; 3 Institute of Pathology, University Medical Center Essen, 45147 Essen, Germany; 4 Department of Visceral Surgery, Sarcoma Center, West German Cancer Center, University Hospital Essen, University Duisburg-Essen, 45147 Essen, Germany; 5 Algorithms for Reproducible Bioinformatics, Genome Informatics, Institute of Human Genetics, University Hospital Essen, University Duisburg-Essen, 45147 Essen, Germany

**Keywords:** GIST, *KIT*, *PDGFRA*, cfDNA, plasma sequencing, NGS, ddPCR

## Abstract

**Simple Summary:**

At some point during long-term treatment, gastrointestinal stromal tumors (GIST) stop responding to treatment due to new genetic changes (mutations). These mutations can be found in the blood of patients using very expensive methods not covered by insurance. We discovered that some mutations can be found in the blood when using cheaper methods that are available in most university hospitals. If the blood was processed immediately, regular blood tubes could be used, and a robot would be better in isolating the genetic information (DNA). Mutations were easier to find in patients with bigger tumors. We compared two different methods to analyze the data. Mutations that were found by both methods were mostly important resistance mutations, but many known mutations from tumor tissue were missed. Taken together, we think that our method cannot replace tumor biopsy or radiologic imaging in GIST, but more precise methods have to be investigated in clinical trials.

**Abstract:**

Circulating tumor DNA (ctDNA) from circulating free DNA (cfDNA) in GIST is of interest for the detection of heterogeneous resistance mutations and treatment monitoring. However, methodologies for use in a local setting are not standardized and are error-prone and difficult to interpret. We established a workflow to evaluate routine tumor tissue NGS (Illumina-based next generation sequencing) panels and pipelines for ctDNA sequencing in an academic setting. Regular blood collection (Sarstedt) EDTA tubes were sufficient for direct processing whereas specialized tubes (STRECK) were better for transportation. Mutation detection rate was higher in automatically extracted (AE) than manually extracted (ME) samples. Sensitivity and specificity for specific mutation detection was higher using digital droplet (dd)PCR compared to NGS. In a retrospective analysis of NGS and clinical data (133 samples from 38 patients), cfDNA concentration correlated with tumor load and mutation detection. A clinical routine pipeline and a novel research pipeline yielded different results, but known and resistance-mediating mutations were detected by both and correlated with the resistance spectrum of TKIs used. In conclusion, NGS routine panel analysis was not sensitive and specific enough to replace solid biopsies in GIST. However, more precise methods (hybridization capture NGS, ddPCR) may comprise important research tools to investigate resistance. Future clinical trials need to compare methodology and protocols.

## 1. Introduction

Gastrointestinal stromal tumor (GIST) is the most common primary mesenchymal tumor of the gastrointestinal tract [1], with an incidence of 10–15 per million per year [2]. GISTs are characterized by activating mutations in the receptor tyrosine kinases c-*KIT* (*KIT*, 80%) or platelet-derived growth factor receptor alpha (*PDGFRA*, ~10%) [3,4], which represent actionable targets for tyrosine kinase inhibitory (TKI) treatment, such as imatinib. Despite long-lasting disease control in patients with metastatic and/or irresectable GIST, the development of imatinib resistance is inevitable in most patients. More than 50% will develop a progressive disease in two years [5]. The most common mechanisms of resistance to TKI therapy comprise secondary mutations in exons 13, 14 and 17, 18 of *KIT* as well as exon 13, 14 and 15 of *PDGFRA*. For *KIT*, this affects the activation loop and ATP binding regions [6], and for *PDGFRA*, mostly the ATP binding region [7]. An additional layer of complexity is added by the possibility of multiple different resistance mutations within one patient [8]. For patients in late lines of treatment a recent trial that used a combination of both tumor and plasma sequencing revealed a landscape of more than 30 different mutations [9]. Drugs that inhibit all known secondary mutations with equal efficacy have not yet been identified [10].

The clinical relevance of detecting resistance mutations—apart from the important scientific aspect—is still unclear. It is of particular interest whether detection of a resistance mutation or combination of resistance mutations could positively impact the choice of drugs or drug combinations. Clearly, single tumor biopsies fail to reveal the full spectrum of resistance mutations within metastasized patients [9]. Therefore, plasma sequencing approaches are much more likely—especially in the metastatic setting—to detect the full degree of genomic heterogeneity of resistance.

A key limitation of plasma sequencing in clinical practice is the lack of reimbursement of in-house assays as well as the high costs of third-party commercial vendors. We therefore aimed to evaluate the feasibility of cost- and time-efficient plasma processing and sequencing in GIST in an academic setting.

## 2. Materials and Methods

### 2.1. Characteristics of This Study

This study was a retrospective analysis of patient plasma samples and related data that were provided by the Westdeutsche Biobank Essen (WBE, University Hospital Essen, University of Duisburg-Essen, Essen, Germany). The project was approved by the institutional Ethics Committee, University of Duisburg-Essen, Germany (12-5279-BO) and was conducted in compliance with the Declaration of Helsinki. All patients provided informed consent. In total, 38 patients were included; all patients had a histologically proven diagnosis of KIT or PDGFRA-mutant GIST. Between November 2010 and January 2017, 103 samples were collected and analyzed.

### 2.2. DNA Preparation

Blood samples (15 mL) were drawn from patients at multiple timepoints during TKI therapy and from healthy donors using Sarstedt^®^ 7.5 mL EDTA monovettes (EDTA) or CELL-FREE DNA BCT^®^ tubes (STRECK^®^), Streck, Lavista, NE, USA. Blood was centrifuged immediately upon receipt at 2500 rpm for 10 min. The supernatant (plasma) was collected and stored at −20 to −40 °C.

### 2.3. Isolation and Quality Control of Free Circulating DNA

cfDNA was isolated manually (ME) using the QIAamp^®^ Circulating Nucleic Acid Kit (Qiagen, Hilden, Germany) or automated (AE) using the Maxwell RSC ccfDNA Plasma Kit (Promega, Walldorf, Germany). The DNA content was measured using the Qubit^®^ Fluorometer (ThermoFisher Scientific, Waltham, MA, USA). DNA quality was analyzed by capillary electrophoresis using the High Sensitivity D1000 ScreenTape^®^ on an Agilent 2100 bioanalyzer (Agilent, Santa Clara, CA, USA). All steps were performed according to manufacturer’s protocols.

### 2.4. Tumor Volumetry

If computed tomography (CT scan) or magnetic resonance imaging (MRI) was conducted at similar timepoints as plasma sequencing was performed, tumor volume was measured using the Centricity RIS System, GE Healthcare, Solingen, Germany. On every 5 mm CT slide, tumor tissue was marked by hand two-dimensionally, and 3D-volumes were calculated.

### 2.5. Routine Next-Generation Panel Sequencing

Different technologies were compared. All runs were conducted on an Illumina MiSeq (Illumina, San Diego, CA, USA) by high throughput massive parallel sequencing of multiplex PCR amplicons. A GeneRead DNAseq Custom Panel V2 and V3 (Qiagen, Hilden, Germany) was used and a library was generated using NEBNext Ultra DNA Library Prep Kit for Illumina (New England Biolabs, Frankfurt am Main, Germany) and data were analyzed using Cancer Research Workbench (CLC Bio, Aarhus, Denmark), Pipeline 1. The panel contained the following genes and exons in brackets: *BRAF* (11, 15), *EGFR* (18–21), *ERBB2* (2, 3, 12, 17, 20, 26), *FGFR1* (3, 7, 13, 17), *FGFR3* (7, 9), *HRAS* (2–4) *IDH1* (4), *IDH2* (4), *KIT* (9, 11, 13, 14, 17, 18), *KRAS* (2–4), *MET* (3, 8, 11, 14, 19), *NRAS* (2–4), *PDGFRA* (12, 14, 18), *PIK3CA* (3, 5, 10, 16, 21), *RET* (7, 10, 11, 13–16), *STK11* (1–9), *TP53* (2–11). Different versions of Qiagen MiSeqDx reagent kits were used: V2 was compared to V3, which—according to manufacturer’s information, “features improved sequencing by synthesis (SBS) chemistry, resulting in higher cluster densities, read lengths, and quality scores compared to previous versions“ [11].

### 2.6. Research Pipeline (Pipeline 2)

For research purposes a Snakemake [12] pipeline: dna-seq-varlociraptor [13] was adapted. Fork of this pipeline with changes, versions and settings of algorithms are accessible on https://github.com/BauerLaboratory/dna-seq-varlociraptor/tree/cfDNA (accessed on 27 September 2022). In summary, the raw panel sequence data were aligned with BWA-MEM [14] to GRCh38 genome reference from Ensembl (release 106). Primer sequences were trimmed (fgbio [15]), bases recalibrated (GATK [16]) and candidate variants (single-nucleotide variants, small insertions and deletions) were called with Freebayes [17] and Delly [18]. Final variant calling was performed with Varlociraptor [19] (events with variant allele frequency ≥0.01). After annotation with Variant Effect Predictor [20], events with moderate and high impact were filtered. Final filtering was conducted with Varlociraptor by controlling false discovery rate at 1%. All mutations detected were classified according to databases COSMIC v96, ClinVar (v20220624), JAX CKB (accession date 12 August 2022).

### 2.7. Droplet Digital PCR

Droplet digital PCR was conducted to detect *KIT* exon 13 V654A and *KIT* exon 14 T670I mutations. Reagents and custom-designed primer/probe mixes were obtained from Bio-Rad Laboratories (Feldkirchen, Germany). Samples of 25 µL containing ddPCR supermix for probes, primer/probe mix (see Table 1 for primer and probe sequences), DNA template and water were prepared in duplicates on a 96-well plate. Every run contained positive and negative controls and no template contamination controls (NTC) controls. Droplets were generated using the Biorad Droplet generator according to manufacturer’s protocol and transferred to a new 96-well plate. PCR was performed on a Bio-Rad T100 Thermal cycler (Bio-Rad, Herkules, CA, USA). PCR temperature steps were enzyme activation at 95 °C for 10 min, 45 cycles of 94 °C denaturation (30 s) and 58 °C annealing/extension (60 s), enzyme deactivation for 10 min at 98 °C and infinite hold at 4 °C. Afterwards, the plate was read by a QX200 Droplet Reader (Bio-Rad), and results were analyzed using Quantasoft Version 1.7.4.0917 software (Bio-Rad).

### 2.8. Cell Lines and Reagents

GIST cell lines established from human GIST have been described previously [21]. GIST cell lines GIST-T1 and GIST430 are imatinib-sensitive. GIST-T1 contains a 57-bp deletion in *KIT* exon 11 [22] and GIST430 has a *KIT* exon 11 deletion (51 bp del V560-Y578). GIST-T1 subline GIST-T1-V654A was genetically modified using CRISPR/Cas9-mediated gene editing [23]. The subline GIST-T1-T670I was generated by selective pressure (imatinib treatment) and was kindly provided by Brian Rubin, Cleveland Clinic, Cleveland, Ohio. Both cell lines are heterozygous for point mutations, leading to 2–3/5 mutant copies per cell.

### 2.9. Statistical Analyses

Statistical analyses were conducted using IBM SPSS statistics 27 (IBM, Ehningen, Germany), and GraphPad Prism version 5.0.0 for Windows, GraphPad Software, (San Diego, CA, USA). Tests performed for statistical correlation were Kruskal–Wallis tests, adjusted for multiple testing, and Mann–Whitney U test as well as Welch tests.

## 3. Results

### 3.1. EDTA Blood Sampling Tubes Require Immediate Processing

**Figure 1:**
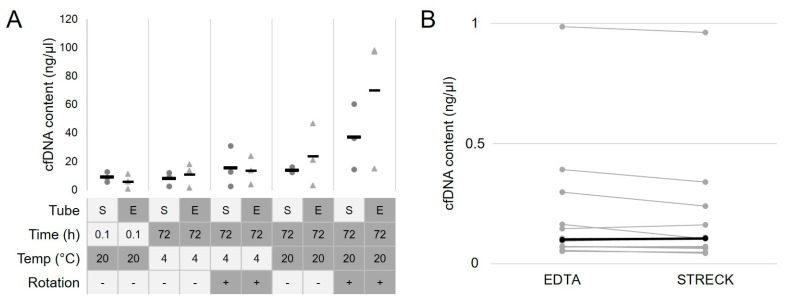
Impact of storage time, storage temperature and physical stress due to prolonged transport of samples on cfDNA concentrations. (**A**) cfDNA concentrations rise over time and physical stress. S: STRECK tubes; E: EDTA tubes; Temp.: temperature. (**B**) cfDNA content of immediately processed blood samples from EDTA and STRECK tubes. Lines connect samples of one patient. Black: median.

In most parts of Germany, plastic EDTA monovettes (e.g., Sarstedt) are used for routine blood withdrawal. Specialized DNA-stabilizing tubes (e.g., STRECK) require adaptors and withdrawal sets unfamiliar to the staff, which may prolong (or even prevent) blood withdrawal. We therefore evaluated the differences in cfDNA content in pre-defined clinical scenarios that could impact the quality of cfDNA (storage time, storage temperature and physical stress due to transport), comparing standard tubes and STRECK tubes as displayed in Figure 1A. Higher cfDNA amounts were assumed as normal cell free DNA contamination. Full blood of three healthy donors was drawn in Sarstedt monovettes (EDTA) and STRECK tubes (STRECK), respectively, and 2 mL each was separately processed before storage at −20 °C. One aliquot of each sample was directly processed, and others were each kept for 72 h at 4 °C or room temperature (RT) with and without slow rotation (1 s^−1^), respectively. Next, cfDNA was extracted manually and cfDNA concentrations were compared. Instant processing consisted of centrifugation within 10 min after blood withdrawal and freezing at −20 °C, and led to a median DNA concentration of 8.9 ng/µL in STRECK tubes and 6 ng/µL in EDTA monovettes, respectively. After 72 h at 4 °C, median cfDNA concentration was stable in STRECK tubes (7.8 ng/µL, factor 0.9) and doubled in EDTA tubes (11.1 ng/µL, factor 1.9). Additional rotation—mimicking physical stress by transportation—increased cfDNA concentrations by 1.7-fold (15.3 ng/µL) in STRECK and 3.2-fold (13.8 ng/µL) in EDTA tubes, respectively. The highest concentrations of cfDNA were observed after 72 h at room temperature (4.6-fold; 23.7 ng/µL), which was further elevated by additional rotation (45-fold; 70.0 ng/µL); in EDTA tubes, this effect was less pronounced than in STRECK tubes (factors 1.8 (13.8 ng/µL) and 5.1 (36.9 ng/µL), respectively, Figure 1A).

Next, blood was drawn from 12 patients into EDTA and STRECK tubes and processed immediately. Following AE, cfDNA concentrations were comparable between both groups (medians of 0.1 ng/µL each, Figure 1B).

### 3.2. Impact of Isolation Protocol on cfDNA Yield and Detection of Mutations

**Figure 2:**
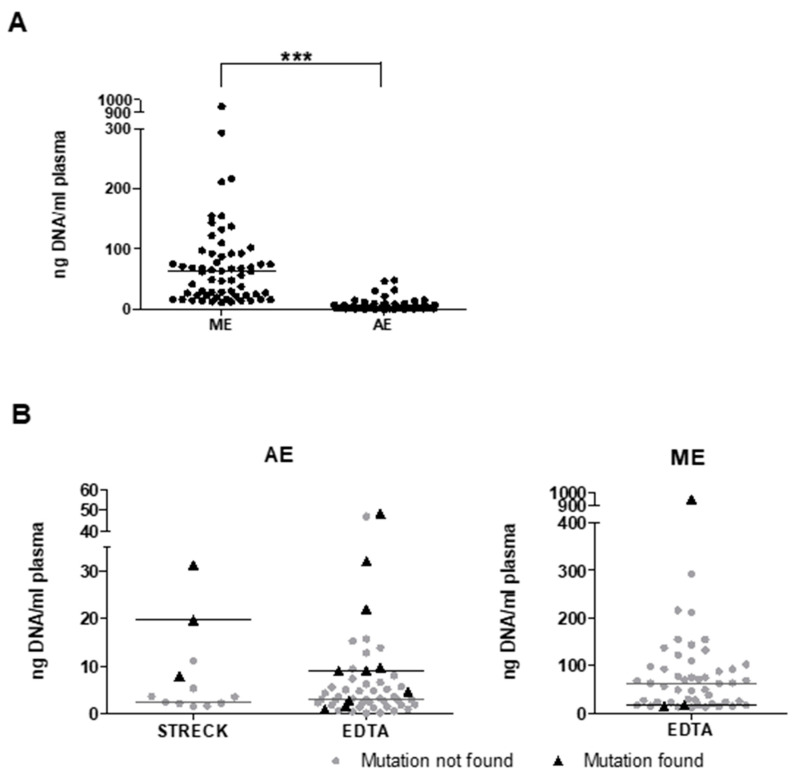
Manual and automated extraction leads to different cfDNA concentrations. (**A**) cfDNA content in automatically and manually extracted (AE; ME) cfDNA of directly processed EDTA-sampled plasma were significantly different (***: *p* < 0.0001). (**B**) Following AE, cfDNA concentrations are higher in mutation-positive samples compared to negative samples. Opposite results were observed in ME cfDNA samples. Horizontal lines: median for specific groups.

Circulating free DNA analyses are not part of routine blood laboratory testing workflows in many institutions. We therefore (retrospectively) compared two commonly used methods in a pathology and research environment to isolate DNA from 133 plasma samples. In 10 patients, a side-by-side comparison was possible. For manual extraction (ME), we used the QIAamp circulating nucleic acid kit, which yielded a median concentration of 62 ng/mL plasma (range: 12 ng to 948 ng; *n* = 62). For automated extraction (AE), the Maxwell RSC ccfDNA Plasma kit was used, resulting in a significantly lower median DNA concentration of 3.55 ng/mL plasma (range: 0.03–48.23 ng/mL; *n* = 70; *p* = 0.00003; Figure 2A). In contrast, we detected known *KIT* or *PDGFRA* primary mutations in only 3/50 samples (6%) following ME, while AE yielded detection of 13/64 (20%), *p* = 0.02. These displayed a trend towards higher median cfDNA concentrations than those without detectable mutations when AE was used (Median STRECK: 19.64 vs. 2.33 ng/mL plasma, *p* = 0.14; EDTA 8.97 vs. 3 ng/mL plasma, *p* = 0.11). For ME samples, DNA concentration was higher when no primary mutations were found (EDTA 17.38 vs. 62.75 ng/mL plasma) (Figure 2B). In these 10 samples, isolated by both methods, concentrations were 1.2–64-fold higher using ME (median ME: 50.1; AE 6.3 ng/mL plasma). Primary mutations were detected in 4/10 using AE and in 2/10 using ME. Importantly, analyses of AE and ME samples were conducted using either V3 or V2 technology, respectively, which may be a confounder for direct comparisons.

### 3.3. Implementation of Panel NGS of Plasma Using Clinical Routine Panels: Sequencing Artifacts as a Pitfall

Panel sequencing of plasma samples using Qiagen MiSeq DX V2 reagent kits by ME revealed a multitude of mutations in *KIT* and *PDGFRA* at low variant allelic fractions (VAF). Primary mutations were detected in 12 samples of seven patients (total: 38 patients, 123 samples) and only one previously identified secondary mutation in a single patient (Figure 2B). To validate low-abundance reads and exclude possible PCR or sequencing errors, healthy donor control plasma was analyzed using two different pipelines. One is used in clinical routine and the other was specifically developed for research purposes. Analysis of 10 healthy donors’ cfDNA (five male, five female) by V2 technology revealed no known secondary resistance mutation in the ATP-binding pocket or the activation loop of KIT. However, several point mutations at low VAF (median 1.09% (0.52–1.89%)) rated pathogenic by fathmmMKL score in the COSMIC database [24] were detected. Recurrent mutations were found in different healthy donors using the routine panel 1: *KIT* V559A in 5/10, N680K and V569A in 2/10, respectively (Supplemental Table S1), at a coverage between 20 and 3280. Of note, we found no mutation that could be simultaneously detected by both pipelines. Due to the high noise detected in these samples using V2 technology, we switched to the Qiagen MiSeqDx Reagent Kit V3 in all subsequent analyses.

To assess the recovery rate of known mutations by NGS and digital droplet PCR (ddPCR), we conducted spike-in experiments with GIST cell lines. Using panel sequencing, healthy donor plasma was spiked with 2% DNA of GIST882 carrying a *KIT* exon 13 mutation and GIST430, carrying a *KIT* exon 11 deletion. The recovery rate was 1.49% and 1.52%, respectively. To determine sensitivity and specificity of ddPCR primers and probes for *KIT* V654A and T670I mutations, parental GIST-T1 DNA was spiked with mutant cell-line DNA. No template controls (NTC) did not show false positives. In parental cell lines, wild-type droplets were detected but no false-positive droplets. Concentrations as low as 0.1 ng mutant DNA could still be detected with V654A and T670I primer-probe pairs. Importantly, using high amounts of healthy control cfDNA (174 ng), no false-positive droplets were detected (Supplemental Figure S1).

### 3.4. Analysis of GIST Patient Plasma Samples

Patient and sample characteristics: We included 38 patients in our analysis. Primary localization was small intestine in 23/38 and stomach in 8/38. One-third of patients carried liver or peritoneal metastases, whereas 18% had both. Plasma samples were mainly obtained from metastatic patients (36/38). The primary mutational status of *KIT/PDGFRA* was known in all patients. Nine of thirty-eight patients had confirmed *KIT/PDGFRA* secondary mutations (Table 2).

### 3.5. Location and Tumor Size May Determine Shedding Rates

**Figure 3:**
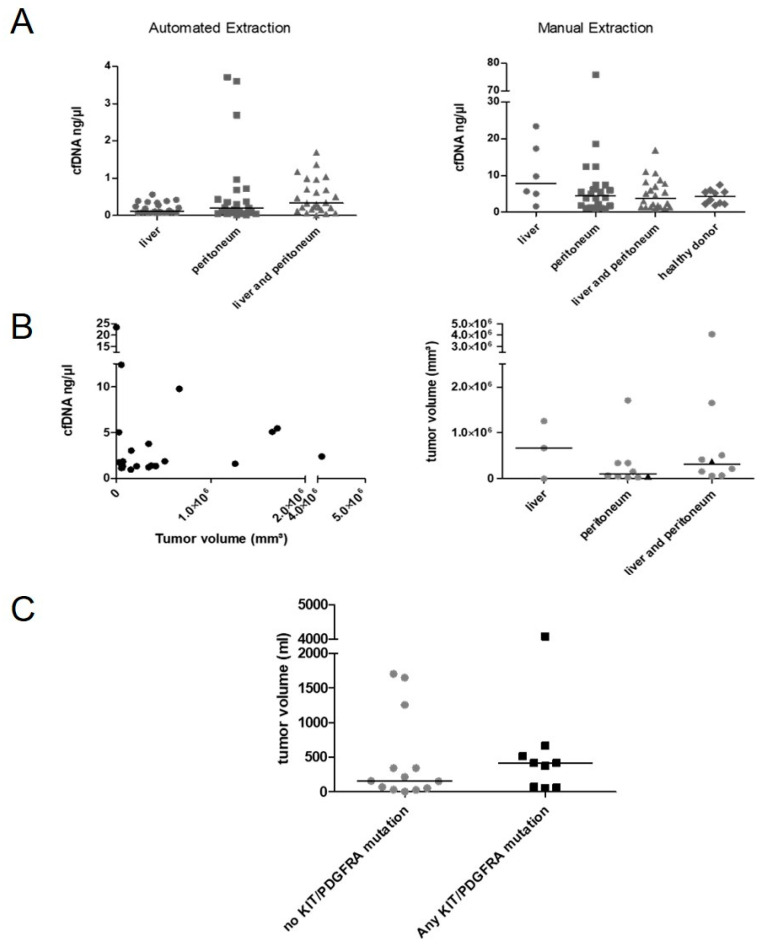
Impact of tumor localization and tumor volume on cfDNA concentration and detection of KIT/PDGFRA mutations. (**A**) Correlation of cfDNA concentration with location of metastasis divided by isolation methods. (**B**) Tumor volume did not correlate with cfDNA amounts (isolated manually). Tumor volume and localization of disease did not correlate. Triangle: primary mutation found. (**C**) Tumor volume was higher in patients with any detectable KIT mutations (not significant). Horizontal line displays median.

To evaluate the impact of primary tumor and metastasis location on cfDNA concentration, Kruskal–Wallis tests were conducted. The median cfDNA concentration following AE was higher in patients with liver and peritoneal metastases compared to liver or peritoneum alone (liver and peritoneum *n* = 25 median 0.33 ng/µL; liver *n* = 22, median 0.12 ng/µL; peritoneum *n* = 23 median 0.2 ng/µL, liver vs. liver and peritoneum: *p* = 0.005, Figure 3A). These relations were not observed in samples isolated manually (liver and peritoneum *n* = 22 median 3.76 ng/µL; liver *n* = 6, median 7.74 ng/µL; peritoneum *n* = 22 median 4.48 ng/µL). cfDNA concentrations of healthy donors were comparable to GIST patients (*n* = 10, Median 4.29 ng/µL, Figure 3A). Tumor volumetry was conducted on CT and MRI scans in 22 cases. Tumor volume did not correlate with ctDNA concentrations or detection of known primary *KIT* mutations (Figure 3B). However, the median tumor volumes were 417 mL in patients with any detectable *KIT* mutation and 158 mL in those without (*p* = 0.3; Figure 3C). The cfDNA concentration was then correlated with radiological response. In AE samples concentrations were significantly higher in patients with progressive disease (*n* = 45, mean 0.53 ng/µL dilution buffer, STD 0.77) compared to stable disease (*n* = 18, mean 0.15 ng/µL, STD 0.16; *p* = 0.003).

### 3.6. Distribution of KIT/PDGFRA Mutations from 133 Plasma Sequencing Analyses

**Figure 4:**
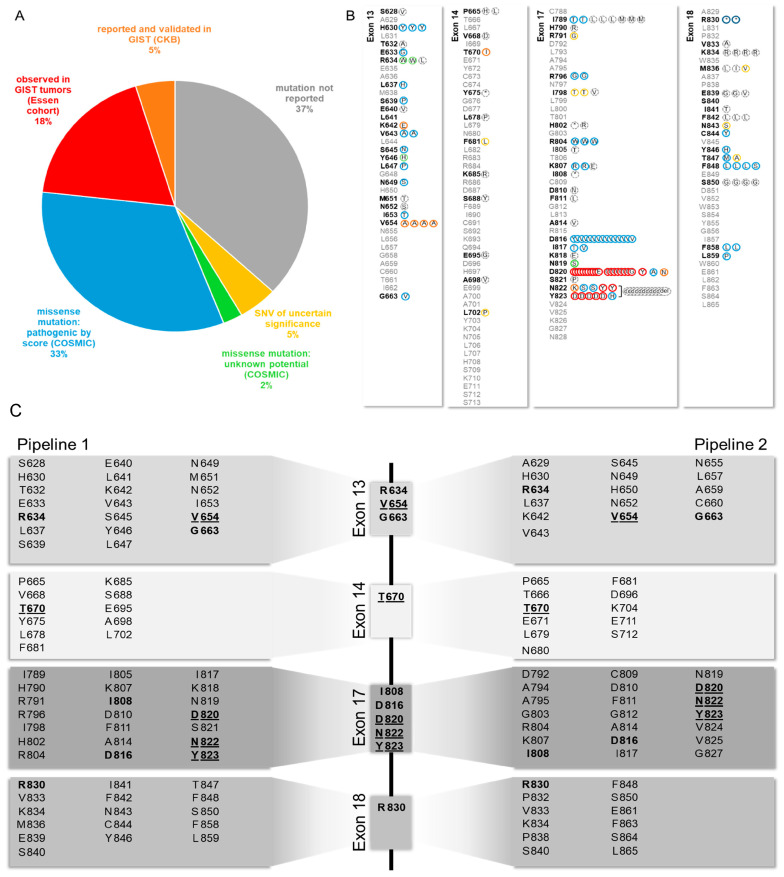
Distribution of KIT Mutations in exons 13, 14, 17, 18 found in patient plasma samples and clinical significance. (**A**) Distribution of clinical significance by manual database search. (**B**) Distribution of KIT mutations found by Pipeline 1, color code adopted from (**A**) Del: deletion, stop codon. (**C**) Analysis of sequencing data using two different pipelines. Pipeline 1: routine pathology pipeline; Pipeline 2: research pipeline. Mutations found by both pipelines displayed in the middle. Bold: found by both pipelines, underlined: Loci for known GIST resistance mutations.

The *KIT* mutations found in our plasma analyses were then compared to tumor samples. Primary mutations were detected in 12/135 samples of 7/38 patients. The detection of deletions of >2 amino acids was significantly lower compared to smaller deletions (5/43 vs. 7/22, respectively, *p* = 0.043). A known secondary mutation was detected in one sample. As resistance mutations do occur in *KIT* exons 13, 14, 17 and 18, only these exons were included in our further analyses. All secondary mutations were categorized according to their clinical significance as stated in databases COSMIC, ClinVar, JAX CKB and our institutional GIST patient database (Figure 4A). Furthermore, 63% of all mutations were reported. Of those, five different mutations were reported and validated in GIST tumors (K642E, V654A, T670I, D820N, N822K) and an additional five (D820E/G/Y, N822Y, Y823D) were found in our institutional database. A further 28 were classified pathogenic and 2 of unknown potential by COSMIC FATHMM score. Seven single nucleotide variants (SNVs) of unknown significance were found and 39 mutations were not reported (Figure 4B).

During this project, bioinformatic pipelines were improved. To evaluate the progress with a focus on detection of sequencing errors and false positives as well as improvement of sensitivity and minimizing false negatives we analyzed the raw data again using the open-access dna-seq-varlociraptor pipeline (Pipeline 2). Using a false discovery rate of 1%, the analysis revealed 182 mutations in *KIT,* of which 53 were detected with both pipelines. Of these mutations, 10 were known primary mutations and one a known secondary mutation. Of note, pipeline 2 detected two primary exon 9 duplications (A502_Y503dup) not detected by the routine pipeline. Seven of the detected mutations in *KIT* exons 13, 14, 17, and 18 were validated GIST resistance mutations. The novel pipeline detected 15 known primary mutations in five patients and 3 known secondary mutations in two patients.

### 3.7. Evolution of Primary and Secondary Mutations of Known Malignant Potential during Therapy

**Figure 5:**
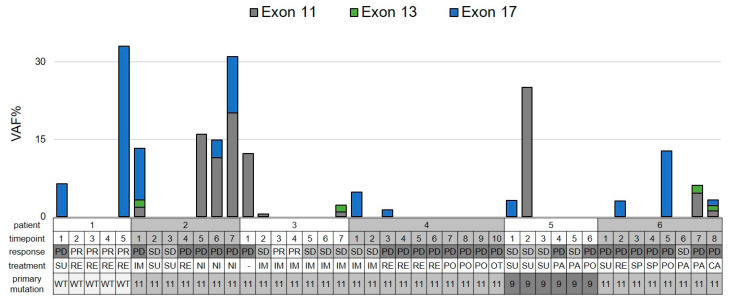
Longitudinal assessment of KIT mutations in GIST patients. Variant allele fractions (VAF) are displayed for each timepoint. Mutations of each exon were grouped. In case of multiple samples per timepoint mean VAF is shown. Y: yes; PD: progressive disease, SD: stable disease, PR: partial remission; IM: imatinib, SU: sunitinib, RE: regorafenib; NI: nilotinib; PO: ponatinib; OT: other; PA: pazopanib; SP: sunintib + ponatinib; CA: cabozantinib; WT: wild-type; 11: exon 11; 9: exon 9.

We then evaluated the detectability of primary and secondary mutations with known malignant potential in GIST in our cohort. To identify timepoints with a higher probability of detection we first analyzed six patients with more than four longitudinal samples (Figure 5). All patients had visible disease at all timepoints in correlating CT/MRI scans and the radiologic response state to the current drug was documented. In this cohort, the number of mutation-positive samples per patient ranged between 20% (2/10) and 57% (4/7). Most mutations were detected at progressive disease state. To validate these results, disease status and detection rate for known resistance mutations were correlated in the whole cohort. The detection rate was 14% (5/37) at stable disease (SD) and 27% (20/73) at progressive disease (PD) state. There was no difference in the proportion of positive samples for these mutations comparing V2 and V3 technology following AE (primary mutations: V2 18% (4/22) vs. V3 21% (9/42); secondary mutations: V2 17% (4/24) vs. V3 15% (7/46)).

Several publications indicate differential sensitivity of *KIT* resistance mutations towards tyrosine kinase inhibitors [25]. We analyzed the detected resistance mutations with regard to TKI resistance profiles (Table 3). All exon 13 mutations resulted in the V654A exchange, whereas exon 17 mutations comprised D820E/G/Y, N822K/Y, N822_Y823delinsKH, and Y823D. The highly TKI-resistant exon 17 D816V mutation was reported separately. In most cases, the outgrowth of mutations fitting the known resistance spectrum was observed except for ponatinib, where mainly exon 17 mutations occurred in 5 of 10 samples.

### 3.8. The Costs for Periodical ctDNA Testing Are Comparable to Radiological Imaging

We tried to evaluate the costs of plasma sequencing compared to regular CT or MRI imaging. Costs were calculated according to the German cost calculation for treatments and diagnostic in patients with state-mandated health insurance “EBM” (einheitlicher Bewertungsmaßstab der Kassenärztlichen Bundesvereinigung), Table 4, [26]. We found that costs using our workflow slightly exceeded costs for regular radiological imaging by 22.29–58.89€ as far as comparisons are suitable.

## 4. Discussion

Plasma sequencing using next-generation sequencing is an emerging companion diagnostic technology for various indications such as BRCA1/2 testing in ovarian cancer, ALK rearrangements in lung cancer and PIK3CA gene mutations in breast cancer patients eligible for treatment with alpelisib [27].

A multitude of clinical trials have evaluated the detection of KIT primary mutations in a neoadjuvant/adjuvant setting in localized disease. A single-center study from Sweden was able to detect known primary mutations in 9/32 patients and 22 of 161 plasma samples. However, this was mainly during surgery or progression but not after complete resection using simple, multiplexed, PCR-based barcoding of DNA for sensitive mutation detection using sequencing (SiMSen-Seq). Primary mutations were detectable in two patients at the time of progression [28]. Using NGS, Kang et al. were able to detect KIT mutations in 13/18 cases before treatment and surgery. Interestingly, in tumors with KIT deletions, only point mutations in the affected gene stretch were detected [29].

In the context of metastatic GIST, cfDNA analyses have been used as a research tool that can help to detect novel resistance mutations but also to better understand the level of genomic heterogeneity of resistance [7,9]. However, treatment sequence in metastatic GIST solely relies on the order of the registration trials and not on detection of specific resistance mutations. Sunitinib and regorafenib have highly differential activity on ATP-binding pocket and activation loop mutations [10] and it is tempting to assume that the presence of these mutations in plasma could be predictive of response and also guide the treatment sequence in GIST.

However, to our knowledge, plasma sequencing has not been confirmed to have a meaningful clinical benefit by prospective clinical trials in metastatic GIST. Moreover, plasma sequencing is not reimbursed by health insurances in most European countries and commercial vendors still remain expensive, with rates of up to USD 5000 for a single analysis with the most advanced technology [30]. The development of academic testing could greatly decrease cost and allow prospective testing in a routine setting.

In this project, we sought to evaluate the feasibility and reliability of plasma sequencing in GIST using pre-existing panel NGS workflows and at the same time establish a cost- and time-efficient protocol for sample processing and biobanking at a European high-volume sarcoma center.

In clinical practice, plasma sequencing remains a rare exception. Outpatient specimen collection for the vast majority of patients includes potassium EDTA tubes, serum tubes, or sodium citrate tubes. Plasma sequencing by commercial providers typically requires cell-stabilizing blood-collection tubes in order to prevent contamination of plasma by cellular DNA from white blood cells [30,31]. These tubes are 100-fold more expensive than regular EDTA tubes (EUR 0.13 vs. 12.76 [32,33]). In addition, special needle adapters are required to use these test tubes with regular blood draw kits in many countries. However, we show that potassium EDTA tubes allow plasma collection for circulating DNA when storage conditions and processing time are well defined. From an economical perspective, the use of EDTA tubes would therefore be desirable. In an experimental setting, we compared blood cell stability in regular EDTA tubes with STRECK DNA blood-collection tubes and found similar amounts of cfDNA when samples were immediately (within 10 min) processed. Notably, when EDTA tubes were kept at 4 °C without mechanical stress, cfDNA remained stable. However, room temperature and mild physical stress increased plasma DNA levels dramatically in EDTA tubes but much less in STRECK tubes. Therefore, the use of EDTA tubes for plasma sequencing should be limited to a setting where immediate processing can be provided or samples can be kept in cool storage without longer transportation (Figure 1). Unfortunately, the small number of samples collected with STRECK tubes in our study does not allow further conclusions, but previous studies have clearly shown the advantages of STRECK tubes [34]. Therefore, when setting up plasma sequencing workflows, the requirement of immediate manual sample processing needs to be weighed against the higher costs of specialized DNA-conserving tubes. Most studies about plasma sequencing in GIST used normal EDTA tubes and processing within 4–24 h [35,36,37,38,39] while blood collected in STRECK tubes was processed within 7 days [31,35,40].

DNA preparation is more commonly performed in molecular pathology laboratories and not in routine laboratories that process blood samples. Plasma sequencing workflows therefore need to incorporate standardized DNA-extraction protocols to ensure comparability. Notably, the majority of studies in GIST used manual extraction [31,35,36,37,39,40,41]. In line with previous studies of van der Leest et al., in our experiments, manual extraction yielded higher rates of cfDNA compared to an automated extraction [42]. This can be explained by the higher concentrations of long DNA fragments after column-based ME. However, ctDNA has been shown to consist of mostly short fragments (90–150 bp) which are enriched in the automated protocols that use magnetic beads for isolation [43]. This may explain why in our study the detection rate of mutant DNA was higher with the AE despite lower yields of cfDNA. Based on these results, we completely switched from ME to AE for future studies.

To evaluate the feasibility of plasma sequencing, we performed panel NGS using routine panels and pipelines designed for GIST tumor pathology. During the course of this project, Qiagen technology was further developed from V2 to V3 to improve sequencing-by-synthesis results to reduce artifacts. The ongoing development of this project over several years led to the use of different NGS panels and analysis pipelines as well as DNA-isolation methods. However, the *KIT* and *PDGFRA* exons covered did not change and there was no difference in the detection rate of primary mutations and secondary mutations commonly found in GIST comparing V2 and V3 technology. Based on these results, we decided to pool all results to analyze the clinical relevance and to identify timepoints with a high likelihood of detection and useful therapeutic readout.

We found that cfDNA concentrations were higher in combined liver and peritoneal metastasis and tumor volume was higher in the any-mutation-positive cohort. However, these results were not significant and CT/MRI measurements did not consider changes in density as recommended in CHOI criteria [44]. Additionally, progressive disease was an indicator for higher cfDNA concentrations and detection of characteristic secondary resistance mutations, consistent with previous studies in GIST [37,40]. As GIST tumors are very heterogeneous, KIT amplification might occur and most mutations are heterozygous, we are not able to conclude on the percentage of circulating tumor DNA fragments within the total amount of cfDNA. This might impact on the correlation of tumor load and cf/ctDNA amount.

Apart from known pathogenic KIT-mutations, routine pipelines detected a multitude of mutations with unknown or uncertain oncogenic potential. We evaluated a novel, open-access research pipeline in order to compare its ability to differentiate sequencing errors from pathogenic mutations. Notably, previously unknown variants called by the research pipeline differed from those detected by the commercial pipeline that was used previously. Mutations detected by both pipelines were mainly known as GIST primary and secondary mutations. Of note, the open-access research pipeline was able to detect primary Exon 9 duplications in two cases that were not detected by the commercial pipeline, as well as three known secondary mutations in two patients (vs. one mutation in one patient by pipeline 1). One study comparing sequencing results of different NGS plasma sequencing vendors (carcinoma subtypes) reported false-positive results mainly at Variant allele fractions (VAF) below 1% and reliable results at VAF > 10%. Most false-positive and false-negative results were technically explained by background noise and bioinformatics filtering thresholds [45]. KIT mutations caused by non-malignant clonal hematopoiesis were not reported [46]. We conclude that an open-access variant-calling pipeline is effectively able to identify pathogenic KIT variants in plasma. However, regular panel sequencing as optimized for tumor biopsies is prone for amplifying KIT variants that are presumably not activating and likely represent background noise. The knowledge about specific resistance-mediating mutations in GIST is crucial to distinguish between real mutations and sequencing errors.

Our data and other publications [37,47] do not support reliable replacement of solid biopsies by ctDNA analysis, yet. As cfDNA sequencing has similar costs to tumor NGS and costs for tumor biopsies are avoided, plasma sequencing may yield an overview of emerging resistance and may thus be clinically useful in GIST. However, results will be less reliable using routine panel sequencing. Multiple vendors offer hybridization capture-based NGS analyses that lead to improved detection rates also in GIST cohorts (NGS Guardant 360: *n* = 162.56% sensitivity, 100% tissue—plasma concordance in metastatic, high disease burden, TKI-refractory patients [31]). Multiple clinical trials in GIST used this method to detect primary and secondary mutations [31,48], so this method is suitable to gain an overview of existing, unknown mutations. However, these methods are expensive and not yet covered by health insurance. While ddPCR is limited to known mutations and by the number of mutations to be detected by multiplexing, it is highly sensitive and specific. A recent presentation has shown that ATP-binding-pocket mutations were a strong negative predictor of avapritinib activity in *KIT* mutant GIST [48]. The vast majority of these mutations, particularly in earlier treatment lines, consists of V654 and T670 substitutions. A prospective trial would ideally evaluate the detection as well as VAF, particularly in drugs with activity against the activation loop using ddPCR. However, the different methods of ctDNA analysis in an academic setting need to be further evaluated in prospective clinical trials to harmonize plasma collection, processing and analysis for cost-effective, comparable and reliable results in GIST.

## 5. Conclusions

In patients with imatinib-refractory GIST, ctDNA analysis can frequently detect resistance mutations, but its clinical value is not yet confirmed. Using pipelines that are optimized for tumor tissue analyses poses the risk of false-positive findings which can be reduced using novel variant-calling pipelines. ctDNA analyses in GIST can be performed in an academic setting but they are labor intensive and will require reimbursement by health insurance in the future. Increasing the accuracy of next-generation sequencing, knowledge of sequencing errors and consecutively improved bioinformatics pipelines will hopefully improve the cost-effective detection of ctDNA mutations in the future. Until then, ddPCR might be ideal for the detection and monitoring of known mutations in routine scenarios, but panel sequencing with improved specificity, such as hybrid capture approaches, will be preferred in the research setting [7].

## Figures and Tables

**Table 1:** ddPCR primer and probe sequences used for ddPCR.

Target	Forward Primer Sequence	Reverse Primer Sequence	Mutant Probe 5′ → 3′ FAM	Wild-Type Probe 5′ → 3′ HEX
***KIT* exon 13 V654A**	TCCTGTATGG TACTGCATGC	GAGAGAACAA CAGTCTGGGT	TGGTGCAGG CTCCAAGTA GATTCGCA	TGGTGCAGG CTCCAAGTA GATTCACA
***KIT* exon 14 p.T670I**	ATGGGAGGCA GAATTAATCT	GATCTTCCTGC TTTGAACAA	CCCACCCTG GTCATTAT AGAATA	CCCACCCTG GTCATTACA GAATA

**Table 2:** Patient and sample characteristics.

AGE (DIAGNOSIS)	Median:	50.5 years (27–76)
GENDER	Male:	25 (66%)
	Female:	13 (34%)
PRIMARY TUMOR LOCALIZATION	Gastric:	8 (21%)
	Small intestine:	23 (61%)
	Rectum:	2 (5%)
	Other:	5 (13.2%)
METASTASIS LOCALIZATION	Liver:	12 (32%)
	Peritoneum:	12 (32%)
	Both:	7 (18%)
	Other	6 (16%)
DISEASE STATUS AT DIAGNOSIS	Localized	22 (58%)
	Metastatic	16 (42%)
DISEASE STATUS AT FIRST PLASMA SEQUENCING	Localized	2 (5.2%)
	Metastatic	36 (94.7%)
PRIMARY MUTATION	*KIT* exon 9	10 (26%)
(TUMOR TISSUE)	*KIT* exon 11	24 (63%)
	*KIT* exon 17	1 (3%)
	*PDGFRA* exon 18	3 (8%)
SECONDARY MUTATIONS	*KIT* exon 11	1 (3%)
(TUMOR TISSUE)	*KIT* exon 13/14	1 (3%)
	*KIT* exon 17/18	7 (18%)
	*PDGFRA* exon 14	1 (3%)
	None:	28 (73%)
NUMBER OF SAMPLES PER PATIENT	1	12 (31.6%)
	2	11 (28.9%)
	3	5 (13.2%)
	>3	10 (26.3%)
TECHNOLOGY	V2	87 (64.4%)
	V3	48 (35.6%)
	- Samples with V2 and V3	21
DNA-ISOLATION METHOD	Qiagen	63 (46.7%)
	Maxwell	72 (53.3%)
BLOOD TUBES	STRECK	14 (10.4%)
	EDTA	121 (89.6%)
	- EDTA and STRECK	12 (8.9%)

**Table 3:** Detection of resistance mutations subdivided by TKI. Expected resistance is shown by color (red: resistant, yellow: intermediate, green: sensitive, grey: unknown).

	Untreated	Imatinib	Sunitinib	Regorafenib	Pazopanib	Avapritinib	Sunitinib and Sirolimus	Ponatinib	Nilotinib	Other
	*n* = 11	*n* = 34	*n* = 14	*n* = 18	*n* = 11	*n* = 3	*n* = 2	*n* = 13	*n* = 10	*n* = 7
Exon 13	0	1	0	0	1	0	0	0	0	0
Exon 17	0	4	1	1	0	0	2	4	5	0
Exon 17 D816	0	2	0	3	0	0	0	0	0	1
Exon 13 and 17	0	1	0	0	0	0	0	0	0	1

**Table 4:** Costs of radiological imaging compared to cfDNA analysis.

Cost per Sample (€)									
EBM								Research Costs	
CT Scan		MRI Scan		CT-Guided Biopsy		NGS Panel Sequencing		ddPCR per Sample and Mutation	
abdominal CT	80.54	abdominal MRI	117.14	biopsy	103.93	STRECK tube	10.50	STRECK tube	10.50
contrast agent	24.03	contrast agent	24.03	DNA Extraction	26.25	DNA Extraction	26.25	DNA extraction (Maxwell)	20.83
infusion	7.45	infusion	7.45	flagfall fee	16.13	flagfall fee	16.13	primers and probes	5.60
				flagfall fee tumorgenetics	42.61	flagfall fee tumorgenetics	42.61	ddPCR reagents	5.12
				Mutation analysis per 250 bp	75.42	Mutation analysis per 250 bp	75.42	ddPCR consumables	6.40
								personnel costs	8.33
total	112.02	total	148.62	total	264.34	total	170.91	total	56.78

## Data Availability

The data presented in this study are available on request from the corresponding author.

## Supplementary Materials

The following supporting information can be downloaded at: https://www.mdpi.com/article/10.3390/cancers14225496/s1, Figure S1: ddPCR spike-in experiments to determine sensitivity and specificity for T670I and V654A primer-probe-pairs, Table S1: Genetic aberrations detected in healthy donor controls.
