# Supplementary material for: Plasma Sequencing for Patients with GIST—Limitations and Opportunities in an Academic Setting
Source: Cancers (Basel). 2022 Nov 9;14(22):5496. doi: 10.3390/cancers14225496 (PMC9688348; doi:10.3390/cancers14225496)

Supplementary Material

ID	*KIT* mutation (AA)	pipeline 1		pipeline 2		Database
		VAF (%)	Cov.	VAF (%)	Cov.	
**F-1**	E490K	1.28	1097			Cosmic
	M552_V559del	1.16	1720			none*
	**V569A**	0.99	1624			Cosmic
**F-2**	F469L	1.03	1455			Cosmic
	N819Y	0.66	1213			Cosmic
**F-3**	N486D	1.29	773			Cosmic
	I817T	0.88	3165			Cosmic
	N680K			4.09	600	Cosmic°
	K704*			2.70	345	Cosmic
**F-4**	**V559A**	1.27	553			Clinvar
	P577L	1.83	546			Cosmic
	C844R			2.82	314	none
**F-5**	**V559A**	1.14	1846			Clinvar
	P573L	1.19	1845			Cosmic
	E671G			3.23	118	none
	S453F			1.79	2263	none
**M-1**	**V559A**	1.89	1536			Clinvar
	P832S			2.63	550	none
**M-2**	**V569A**	1.09	2568			Cosmic
	S688*			3.00	523	none
**M-3**	**V559A**	0.59	1703			Clinvar
	A629V			1.80	2826	none
	L679*			3.38	568	none
**M-4**	R586*	0.52	3280			Cosmic
	K818E	0.85	1760			Cosmic
	I817N			2.56	3450	none
**M-5**	**V559A**	0.81	1235			Clinvar
	E562G	1.12	1250			Cosmic
	W582*	1.01	891			Cosmic

Suppl. Table 1: Panel sequencing (V2) of manually extracted healthy donor cfDNA and subsequent use of different pipelines for analysis led to detection of various different genetic aberrations in KIT. Recurrent aberrations by one method are marked in bold letters. F: female, M: male. °mutation found in GIST, * stop codon.

Supplemental Figure S1

Supplemental-Figure S1: ddPCR spike-in experiments to determine sensitivity and specifity for T670I and V654A primer-probe-pairs. **A:** No template controls (black: empty/ double negative droplets), 2 wells merged **B:** parental cell line GIST-T1 (green dots: wildtype-positive droplets), 2 wells merged. **C:** spike-in of mutant cell line into parental cell line (blue dots: mutation-positive droplets), 2 wells merged **D:** healthy donor cfDNA in high concentration, no detection of mutation-positive droplets, 4 wells merged. DNA concentrations are sum of all wells displayed in the graph.

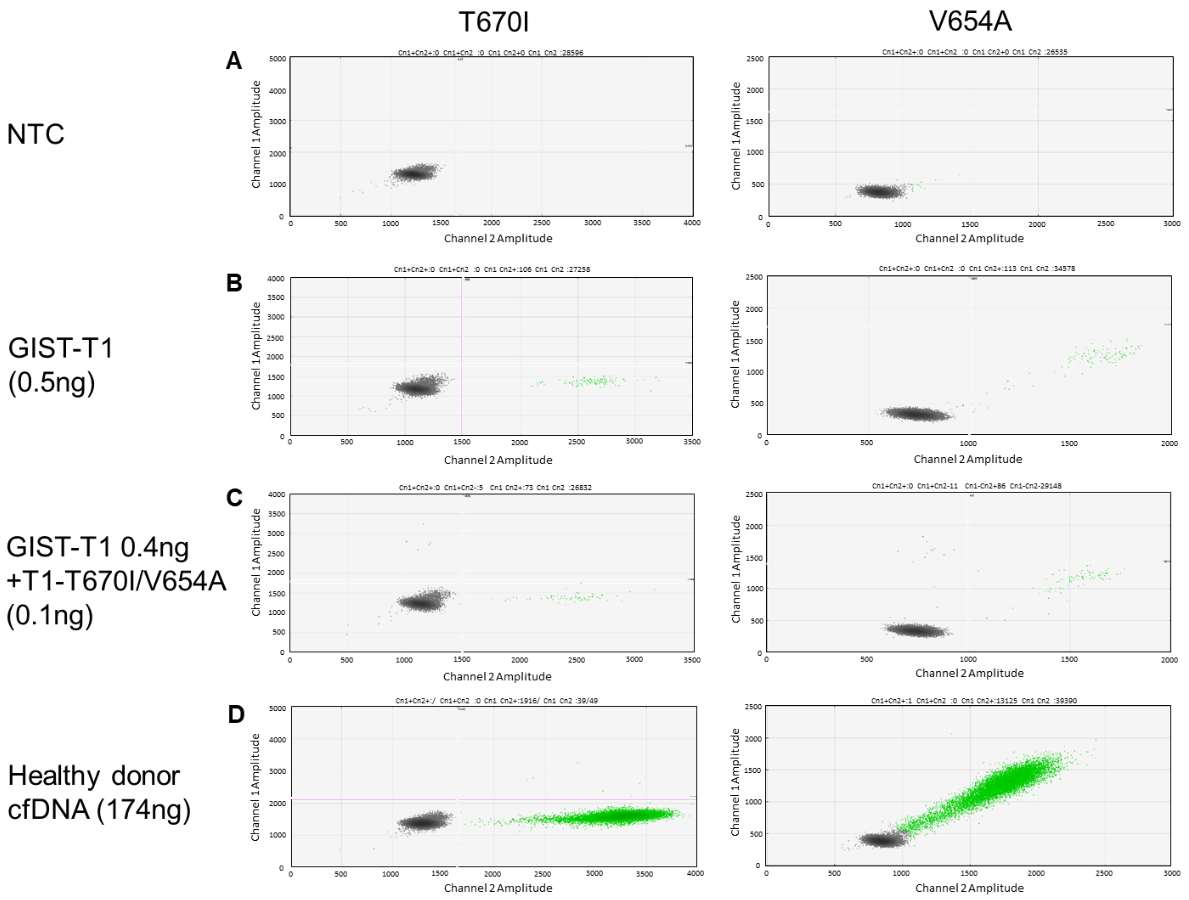

Supplement: Supplementary file 1 [file cancers-14-05496-s001.zip › cancers-1969013-supplementary.pdf]
